# The Urine in Typhoid Fever

**Published:** 1894-06-02

**Authors:** 


### Medical Progress and Hospital Clinics.

[*The Editor will be glad to receive offers of co-operation and contributions from members of the profession. All letters should be addressed to* The Editor, The Lodge, Porchester Square, London, W.]

### THE URINE IN TYPHOID FEVER.

Much attention has of late years been devoted to this subject, chiefly because Ehrlich in 1882 described his diazo-reaction. He claimed that in typhoid fever the addition of diazo-benzene—sulphuric acid, in the presence of ammonia—to the urine, formed, with some unknown peculiar pathological substance, a body of a characteristic red colour, which was best seen in the foam produced on the top of the mixture. To perform the test two solutions are necessary. The first is a five per cent. solution of hydrochloric acid in a saturated aqueous solution of sulphanilic acid, and secondly a half per cent. solution of sodium nitrite. When required for use 40 cc. of the former and 1 cc. of the latter are mixed, and by the action of the hydrochloric acid on sodium nitrite nitrous acid is set free, and this, combining with the sulphanilic acid, gives rise to diazo-benzene—sulphonic acid. Erhlich states that equal parts of this mixed solution and the suspected urine should be shaken together, after which strong ammonia is added. If the test can be obtained a characteristic pink tint is seen on the foam, and at the point of meeting of the ammonia and the mixture containing urine a dark red ring appears, and on shaking the tube the whole of the mixture becomes of a dark red colour.

After twelve years' trial of this test it is possible to form some idea of its efficacy. Several observers have tried it in numerous cases of persons suffering from all sorts of diseases. Ruhmeyer has published a collection of 260 cases, in which the urine was tested 2,750 times, and he concludes that the test is very valuable, although he occasionally found it in cases of chronic nephritis, caries, pyæmia, scarlet fever, pleurisy, tubercular meningitis, pneumonia, primary pulmonary actinomycosis, carcinoma, sarcoma, and tuberculosis. The most characteristic reaction was, however, obtained in pulmonary tuberculosis and typhoid fever. Eduans comes to the following conclusions, after making a series of 600 examinations of the urine. It was absent in only two out of 130 cases of typhoid fever, its usual duration was thirteen days. Its intensity bore no relation to the temperature nor to the seriousness of the case. He sums up by saying that it may help to the diagnosis of typhoid fever, but that it is not of a very high diagnostic value.

Perhaps the most complete paper on the subject is one by Van Noorden, who not only gives us a full literature of the subject, but also tells of the results obtained by testing the urine of the patients in Gerhardt's clinic. In five years he only found the test absent in one undoubted case of typhoid fever, so that it may be regarded as a very constant symptom; but unfortunately this reaction may be obtained in many other diseases. Thus he found it in advanced heart disease, cirrhosis of the liver, carcinoma, meningitis, erysipelas, pneumonia, scarlet fever, diphtheria, and all forms of tuberculosis. Thus, while it may confirm the diagnosis in cases in which the diagnosis is easy, it does not help us much in doubtful cases.

Hewetson, working in Professor Osler's clinic, looked for the re-action in 196 cases, and found it in 136; but he remarks that as it may disappear by the end of the second week, it is very highly probable that the re-action would have been found in a larger proportion of cases if it had been possible to test them during the whole of the illness. Of fifty cases which were particularly carefully observed the reaction was present in 77 per cent. during the first week, in 67 per cent. during the second, in 47 per cent. during the third, and in 17 per cent. during the fourth. Its duration varied very much; sometimes it only lasted a few days. It might be met with during a relapse, and it might persist during the whole of an attack. The intensity seemed to bear no relation to the gravity of the case nor to the height of the fever. It was found in very many other diseases, chiefly tuberculosis, measles, scarlet fever, and pneumonia. Actually 34 per cent. of the cases of tubercle showed it, and it was very commonly present in acute miliary tuberculosis.

On the whole we may therefore conclude, after the profession has had abundant trial of this reaction, that it has very little diagnostic value.

The other point which has also received much attention lately, is the toxic property of the urine of patients suffering from typhoid. It is well known that healthy urine is toxic, and Roque and Weill investigating the urine of typhoid patients in whom no treatment was adopted, find that the toxicity of the urine is much increased, being double that of health, but that this poisonous property of the urine is independent of the temperature, and remains increased during the whole of the fever, and also during the first part of convalescence. With regard to the cases in which antipyrin was used, they come to the conclusion that the drug appears to act as an antiseptic, the urine loses all its toxicity without there being any evidence of the retention of toxic bodies in the system, but when the drug is stopped the toxic bodies again appear in the urine, and are excreted in large quantities during convalescence. Patients treated with cold baths excrete enormous quantities of toxines during the cold bath treatment, but proportionately less during convalescence. These results are very interesting, and may in part explain the good results that have been derived from the cold bath treatment.

With regard to this question of toxines in the urine, it is interesting to note that the importance of getting rid of toxines from the body is gradually getting well known, and was admirably illustrated at the meeting before last of the Clinical Society of London, when Dr. Lander-Brunton and Mr. Watson Cheyne read an account of a case in which the evacuation of toxines from the intestine in a case of intestinal obstruction quickly led to marked improvement, and Dr. Hale

White related a case in which the evacuation of a quantity of fæcal pus from the pleural cavity led instantly to a great improvement in the patient's general condition, and the increased strength of the pulse was very noteworthy.

